# “Engaging with birth stories in pregnancy: a hermeneutic phenomenological study of women’s experiences across two generations”

**DOI:** 10.1186/s12884-017-1476-4

**Published:** 2017-09-04

**Authors:** Lesley Kay, Soo Downe, Gill Thomson, Kenny Finlayson

**Affiliations:** 1 Kingston and St. George’s Joint Faculty, Health, Social Care and Education, St. George’s Campus, Cranmer Terrace, Tooting, London, SW170RE UK; 2 Research in Childbirth and Health (ReaCH) Group, University of Central Lancashire, PR12HE, Preston, Lancashire UK; 3 Maternal and Infant Nutrition and Nurture Unit, University of Central Lancashire, PR12HE, Preston, Lancashire UK

**Keywords:** United Kingdom, Pregnancy, Parturition, Childbirth, Midwifery, Personal narratives, Qualitative research, Hermeneutics

## Abstract

**Background:**

The birth story has been widely understood as a crucial source of knowledge about childbirth. What has not been reported is the effect that birth stories may have on primigravid women’s understandings of birth. Findings are presented from a qualitative study exploring how two generations of women came to understand birth in the milieu of other’s stories. The prior assumption was that birth stories must *surely* have a positive or negative influence on listeners, steering them towards either medical or midwifery-led models of care.

**Methods:**

A Heideggerian hermeneutic phenomenological approach was used. Twenty UK participants were purposively selected and interviewed. Findings from the initial sample of 10 women who were pregnant in 2012 indicated that virtual media was a primary source of birth stories. This led to recruitment of a second sample of 10 women who gave birth in the 1970s-1980s, to determine whether they were more able to translate information into knowledge via stories told through personal contact and not through virtual technologies.

**Results:**

Findings revealed the experience of ‘being-in-the-world’ of birth and of stories in that world. From a Heideggerian perspective, the birth story was constructed through ‘idle talk’ (the taken for granted assumptions of things, which come into being through language). Both oral stories and those told through technology were described as the ‘modern birth story’. The first theme ‘*Stories are difficult like that’,* examines the birth story as problematic and considers how stories shape meaning. The second ‘*It’s a generational thing’,* considers how women from two generations came to understand what their experience might be. The third *‘Birth in the twilight of certainty,’* examines women’s experience of *Being* in a system of birth as constructed, portrayed and sustained in the stories being shared.

**Conclusions:**

The women pregnant in 2012 framed their expectations in the language of choice, whilst the women who birthed in the 1970s-1980s framed their experience in the language of safety. For both, however, the world of birth was the same; saturated with, and only legitimised by the birth of a healthy baby. Rather than creating meaningful understanding, the ‘idle talk’ of birth made both cohorts fearful of leaving the relative comfort of the ‘system’, and of claiming an alternative birth.

## Background

The birth story as *‘a feminine, woman-to-woman legacy’* has been understood as a crucial source of knowledge about childbirth for mothers [1, 2]. There is a suggestion that such storytelling arises from an intuitive urge to share important events in our lives; our detailed account being an ‘*ancient method of coming to terms with our own experience’* [3]. Telling stories about birth may enable women to assimilate their memories of this transformative event [4]. This may be especially pertinent if the reality of a woman’s experience is not as she imagined; telling stories may have a healing or cathartic effect for women whose experience has been contradictory, disappointing or traumatic [5].

Articulating the birth experience into a story gives it structure*;* once the experience has structure there is potential for meaning to be determined and emotional responses considered [6]. Walsh suggests that women should have the opportunity to find meaning and purpose in the act of giving birth [7]. Savage agrees arguing that birth is not just about delivering babies but is about women’s lives; a woman’s experience potentially having long term implications for her sense of self-efficacy and her ability to form relationships with others, including her infant [1, 8].

In the exchange that takes place when a story is told, the ‘*learner*’ may reconstruct knowledge amassed from the story [9]. During this learning process there is a potential opportunity to lessen fears about birth and to amass a sense of control but there is also an opportunity to increase fears and make women feel powerless [10]. In positive stories women may hear of strength and power in birthing and may be assured of the capacity of women to birth physiologically; conversely in negative stories women may associate birth with difficulty and suffering and the process with risk and fear [1].

Contemporary literature relating to childbirth appears to be primarily concerned with issues of safety and risk [11, 12]. A smaller number of studies consider the meaning of birth and its impact on women’s lives [8, 13]. Of these few question how women understand the meaning of birth prior to the experience and there is little consideration of the influence that other women’s stories may have on primigravid women’s understanding of birth.

This study was unique in that it considered how women from two different generations came to understand birth in the milieu of other women’s stories. The study started with the idea that birth stories must *surely* have a positive or negative influence on listeners and those stories must have the potential to steer women either towards or away from medical and/or midwifery-led models of care.

An understanding of ‘storytelling’ where stories are personal to the storyteller, are spoken and heard in a classic model and are told on a one-to-one basis [14] was the starting point. Initial findings, however, revealed that women share and utilise a variety of different story mediums to prepare for birth. The study reinforced the notion that stories can be fashioned from a single medium or can stretch across a myriad of mediums. For the purposes of this study a story became *‘simply a thing, any media object, which demonstrates…a clear (story arc) sequence*’ and which has the capacity to engage its audience [14]. This notion was identified as the ‘modern birth story’.

## Methods

### Aim

To describe and consider how engaging with stories of birth influenced expectations and experiences of childbirth for two generations of women. For this purpose, birth stories encompassed personal oral stories as well as media and other representations of contemporary childbirth, all of which had the potential to elicit emotional responses and generate meaning in the interlocutor.

### Design

An interpretative hermeneutic phenomenological approach underpinned the study design. Phenomenology studies the way things materialise in our experience as well as the ways we experience things in the world around us [15]. In phenomenological research the researcher seeks to understand human experience by exploring the lived experience or ‘life world’ of the participants [16]. Hermeneutics is a way of thinking about our being, can describe human understanding, and provides a means of questioning existing notions of truth, reason, and knowledge [17]. Modern hermeneutics explores human phenomena by studying human experience as if it has a linguistic and textual structure; that is it tries to ‘read’ human practices, affairs and circumstances in ways that create understanding [18]. Adopting a hermeneutic phenomenological approach enables the researcher to shed light on a phenomenon by a process of ‘*contextualisation and amplification*’ [19].

### Reflexivity

All attempts at understanding in hermeneutic phenomenology start with the researcher as an active participant and involve a moving back and forth between the self, the data and the literature. In phenomenological research the researcher takes with them a number of presumptions which govern the enquiry and potentially what can be discovered [20].

To make sense of the meanings buried in the stories of the participants the primary researcher (LK) explored her presuppositions and understandings of the phenomenon. An interview was conducted with two members of the team and this highlighted a fundamental belief in the ability of most women to birth physiologically, a passion to foster positivity in relation to birth and a belief that birth experiences can permeate the whole of a woman’s life. This explication allowed the researcher to be open with the ‘other’ (the participants); this awareness, along with that gathered through the completion of a reflexive diary and via the sharing and discussion of transcripts with members of the team, contributed to the interpretation [21].

### Recruitment and participants

A purposive sampling method (and snowballing by word of mouth) was used to recruit 20 women; 10 women who were expecting their first baby in 2013 and were registered on a ‘National Childbirth Trust’ (NCT) course and 10 women who had birthed in the 1970s-1980s and were members of the ‘National Federation of Women’s Institutes’ (NFWI) and the ‘Cambridge Businesswomen’s Network’ (CBN). The NCT, the UK’s largest childbirth charity, was targeted to try and ensure that the women had an interest in the significance of birth and provided a finite population from which to recruit. The NFWI and CBN were targeted to ensure (as far as possible) that the women would have a similar socioeconomic status as the women recruited from the NCT. There is a limited amount of data on NCT members but in a 2011 report, NCT members responding to a survey were described as 94% white, 50% early thirties and 86% with a higher degree [22].

### Data collection

Data was collected via face-to-face and telephone interviews. The interviews were audio-recorded, took between 45 to 90 min to complete and were transcribed verbatim. The interviews were non-structured and involved asking ‘hermeneutical questions’ as determined by Ironside [23]. This meant that the questions asked steered away from emotions and feelings instead using questions such as ‘*what does it mean to have experienced…?*’ Questions such as ‘*how do you understand your impending birth’?* And ‘*how did this story make you feel about your pregnancy?*’ were used to encourage participants to share their experiences.

### Data analysis

The interpretation was informed by Smythe’s phenomenological approach; the approach allows the writer to *‘bring the unsaid into an open space’* by utilising a series of questions to stimulate thinking, writing and showing [24].

Each of the transcripts was read by all members of the team; each considering their response, recognising the phrases that leapt out and seeing connections. The primary researcher ‘*crafted*’ a story from each participant’s interview (by finding the story within the transcript as advocated by Crowther et al.) and then interpreted from her perspective the meaning that lay behind the saying [25]. LK considered what the literature had to say and included the thoughts and significances as seen by the rest of the team. LK then interpreted the story, in response to growing understandings, letting the themes emerge and deciding on the best stories to show a theme. LK drew on phenomenological notions to inform her thinking and finally formed an argument articulating the meaning of the phenomenon.

### Rigour and trustworthiness

Rigour was ensured using the adapted framework devised by de Witt and Ploeg [26]. Table 1 illustrates its application.

**Table 1:** ‘Practical Expressions of Rigour’ [26]

Practical expression of rigour	Characteristics of expression	Application in study
Balanced integration	Intertwining of philosophical concepts in methods and findings Balance between the voices of participants and philosophical explanation	Philosophical framework described in methods and applied in findings The researcher’s voice, that of the participants and phenomenological notions give voice to the experience
Openness	Systematic, explicit process of accounting for decisions made throughout study	Transparent audit trail of decision making in relation to design and evolution of the study
Concreteness	Usefulness for practice of study findings	Implications for practice and study limitations discussed
Resonance	Experiential or felt effect of study findings upon the reader	Resonance acknowledged at conference presentations
Actualisation	Future realization of the resonance of the study findings	Acceptance of papers at peer reviewed conferences

### Ethics

Ethical approval was obtained from the Science, Technology, Engineering, Medicine and Health (STEMH) Ethics Committee in April 2012 (phase one), January 2014 (phase two) and an amendment was approved in November 2014 (project number: STEMH 005). Written consent was taken before the interviews were commenced, confidentiality of the data was guaranteed and anonymity of the participants ensured by the use of pseudonyms. Table 2 illustrates the characteristics of the interviewees.

**Table 2:** Characteristics of Interviewees

Demographic group	Phase one (*n* = 10)	Phase two (n = 10)
Dates of interviews	October - December 2012	November 2014 - January 2015
Recruited from	NCT (*n* = 10)	NFWI (*n* = 8) CBN (*n* = 2)
Age Range	27 - 39	52 - 67
Average age	30	57
Ethnicity	White British (*n* = 8) Chinese (*n* = 2)	White British (*n* = 10)
Place of residence	East of England (*n* = 10)	East of England (*n* = 4) North East (*n* = 3) Yorkshire and the Humber (*n* = 2) South East (*n* = 1)

## Results

This study suggests that birth stories are a significant part of the landscape of birth for childbearing women. An understanding of the way of being-in-the-world of birth, as illustrated by the participants’ stories and interpreted according to Heidegger’s philosophy, precedes a discussion of the themes putting these in context for the reader.

### Being-in-the-world of birth

Women’s pre-understandings about childbirth are rooted in their experience of ‘*being-in-the-world’* of birth; women experience aspects of this world in relation to other people in that world. Often these people are members of a woman’s family, her close friends and acquaintances. In their pregnancies women find themselves in a world that appears to operate in a certain way and where certain things have already shown up as important. Heidegger describes this as ‘*thrownness*’, explaining that Dasein (the human kind of being) is ‘*thrown’* into its *‘there’* [27]. As ‘thrownness’, Dasein finds itself already in a certain moral and material, historically conditioned environment [27].

*‘Thrown’* into the world of birth, women are faced with an array of options and choose possibilities of action that are conditioned by their enculturation into the practices of their specific childbearing community. Thrown into this world women attune themselves, creating their existence in terms of what they see as possible. As ‘*everyday being-with-one-another’* women are dependent on others and *‘they’* inconspicuously dominate the way to be [27]. Heidegger’s concept of the ‘*they*’ alludes to the particular community into which we find ourselves thrown. It is a ‘*primordial ‘publicness’ that serves as a shared basis for everyday understandings’* [28]. In our everyday lives we do what ‘*one*’ does according to the norms laid out by the ‘*anyone*’ of which we are a member.

Not only are women *‘thrown’* into a particular world of birth they also *‘fall’* into the dialogue and speech of that world (much of which may be ‘*groundless’* and yet appear to be *‘authoritative’*). This means that what is shared and heard about birth in everyday conversations and via the popular media makes a difference to what women understand about birth. Heidegger uses the term ‘*Gerede’* (‘*Idle talk’*) to describe the way of speaking within our shared world [27]. *‘*Idle talk’ is: *‘the form of intelligibility manifest in everyday linguistic communication - average intelligibility’* [29]. Steiner refers to the phenomenon as ‘*vacuous high gossip*’ suggesting that people use this way of communicating as a ‘*pretence*’; a means of appearing ‘*busy*’ and ‘*well-informed’* in their everyday lives [30].

### Themes

#### ‘Stories are difficult like that’

This theme highlights the problematic nature of the stories being told and the difficulties of sharing stories. The sub-themes of ‘*horror stories*’, ‘*media portrayal’* and *‘too perfect and wonderful: being economical with the truth’* explore the social and cultural norms around the content and sharing of stories.

#### ‘Horror stories’

The women birthing in the present day concentrated on the negative stories they had heard. Stephanie spoke about the, ‘*oh my god it’s so painful’, it’s just so painful’,* kind of stories, elaborating with the comment, *‘You don’t get anyone who says, ‘it’s brilliant, calm, relaxed’. You just get these horror stories’.* Most of the women seemed accepting of the negative stories; Isabel went as far as to say, *‘I have a morbid fascination with them’*. For Stephanie it seemed another facet of modern life:

*‘What I found, it’s like you can go and buy something from Amazon, and you’ve got reviews. Some people will put up the good reviews but most of the people who are making the effort to put a review on is because it’s negative‘.*

The women who birthed in the 1970s-1980s similarly reported that negative stories were shared more readily and frequently than positive ones. Emma, for instance, said that she could not remember any specific stories but that ‘*you always tend to get the horror stories don’t you?’* Carole, who suffered from *‘toxaemia’* whilst pregnant, said her mother ‘*terrified’* her with stories about people she knew who had had the same problem and whose pregnancies had not been successful.

What is shared and heard about birth in their everyday life makes a difference to what women understand about birth. Irwin describes how our *‘being-in-the-world comes through story and through technology*’; helping us to *‘solidify culture’* and share knowledge [31]. Heidegger explains that when we communicate we talk and make claims and in doing so we do not ‘*so much understand the entities which are talked about’* but rather that we concentrate on what is claimed about the entity [26]. We accept what is claimed, simply because it is said, and we pass it on, further disseminating the claim. The result of this is that talk becomes nothing more than ‘*complete groundlessness*’ [26].

#### Media portrayal

Without exception the women birthing in the present day talked about media representations of birth and all mentioned watching (or not watching) a popular television programme. The women seemed clear that the stories shown were chosen for a reason, for instance Ruth said:

‘*Obviously, I know they only pick certain stories to go on TV, they’ve got to make good TV so that’s why they do it’.*

Isabel said:

*‘In 99% of the cases there’s a woman who is lying on the bed in agony giving birth… a lot of them are forceps deliveries, and a lot don’t look particularly calm and enjoyable…but it makes good TV I guess’.*

For the women who gave birth in the 1970s-1980s media portrayals of birth were few and far between. The women described how they learnt about birth from conversations with other women, antenatal classes and books. Two of the women recalled seeing a video of a birth at an antenatal class but neither felt it had been very valuable. Meg was the exception saying that she had a ‘*romantic idea*’ of what it might be like to have a baby and that afterwards she felt:

‘*Quite bitter and twisted that people hadn’t been more honest about how difficult it could be, you know, to give birth*’.

When asked about contemporary television programmes Meg argued that ‘*they probably are a more accurate representation than anything I was shown’* arguing that she would have found them useful.

In a world where the public way of understanding birth (the ‘*drama*’ of birth as described by Ruth) is disseminated so widely women may find themselves *‘taken in a peculiar direction and….absorbed in the immediate, in fashions, in babble’* [32]. Being caught up in the ‘hype’ around birth could mean that women understand ‘*what is said-in-the-talk’* but that what the talk is about is *‘understood only approximately and superficially’* [27]. The inference being that women in today’s ‘world of birth’ may be approaching childbirth with an average understanding of the *claims* about birth as opposed to a genuine understanding of birth itself.

#### ‘Too perfect and wonderful; being economical with the truth’

For many of the women positive birth stories were an anomaly and *‘too perfect and wonderful’* to be believed. Ruth who was pregnant in 2012, for instance, was told positive stories by her yoga teacher, but effectively dismissed them saying:

*‘They’re all you know, amazingly positive experiences and, you know….. I don’t know if I fully believe that she hasn’t taken out some of the bits and pieces. I’m not sure’.*

Ruth was used to hearing stories about interventions and about women birthing on a bed in ‘*excruciating pain’*; she clearly thought that the yoga teacher was imposing a certain perspective on the stories. The result, she said was that the stories were ‘*a bit wishy-washy*’; the inference being that when engaging with a birth story Rebecca wanted it to grab her attention but more importantly she wanted it to fit within her frame of reference. Stephanie said that she would like to hear more positive stories of birth, as opposed to the *‘horror’* stories she has heard countless times. Despite wanting to hear more positive stories Stephanie was dubious when she recalled a positive story, saying that:

*‘Everything was kind of real gushy…and I was like ‘yeah, I’m sure it wasn’t because it was just…everything was too perfect and wonderful?’*

After hearing countless ‘horror’ stories and being exposed to dramatically edited television representations of birth it is hardly surprising that positive stories were not always accepted as ‘real life’; they were at odds with the majority of stories in circulation and with women’s perceived understandings of birth. More than that because of human beings ‘*everydayness*’ and ‘*absorption*’ in the world what is extraordinary (the ‘horror’ of birth described in a story) is made ordinary through familiarity; the appearance of ‘horror’ in a story accommodated and then made invisible by that accommodation, with other interpretations effectively being ‘closed off’ [27].

Whilst the women giving birth in the present day were sceptical about *hearing* positive stories, the women who birthed in the 1970s-1980s spoke about the fact that they were loath to *tell* positive stories for fear of making others feel bad. Penny for instance said that she was being careful about ‘*pushing the whole breastfeeding thing’.* For her the experience of breastfeeding was ‘*magical’* and she wanted to promote it but she was conscious that people might have difficulties with feeding and might feel they had failed if they weren’t successful.

#### ‘It’s a generational thing’

This theme explores how women from two different generations came to understand what their experience of birth might be. The sub-themes of ‘*it was all a bit shrouded in mystery: we let it take its course’, ‘this generation nothing’s private to them’* and *‘information seeking and saturation*’ explore the information seeking behaviours of the women and consider what mattered to the two cohorts of women when anticipating birth.

#### ‘It was all a bit shrouded in mystery: We let it take its course’

The women who gave birth in the 1970s-1980s had little knowledge and understanding of birthing in their pregnancies. Sandra said that other than being told by the midwife that ‘*it will hurt, expect it to hurt’* she had no other knowledge but that instead ‘*it was all a mystery until you actually gave birth’*. The women talked about not really having a voice in their care and indicated that they looked to the health professional for guidance. For these women care was something provided by an ‘expert’ who made decisions for them. As passive recipients of care Pamela said that ‘*we tended to just accept what we were told’* and ‘*went through the procedures that were suggested’.*

For many of the women ignorance was ‘bliss’; Carole explained that if she had had access to the internet when she was pregnant and had researched some of the complications she would have *‘terrified’* herself. Sophie said that there was ‘*an element that I didn’t really want to know*’ primarily it seemed because she did not want to know about the ‘*things that could go wrong’.*

Women in this era were birthing at a time where the norm was to birth in a hospital in a ‘system’ where birth was only considered normal in retrospect and where interventions were accepted as part of routine care; pregnant women were treated as hospital ‘patients’ under the care of an obstetrician and their care was typically focused on the needs of the institution as opposed to the needs of the individual [33]. Paula’s experiences are a good example of this; she talked about going into labour on New Year’s Eve and about being put on a drip to speed up the contractions, observing:

*‘Because she was born on New Year's Eve and I was thinking is this more about the time of year than actually about me, you know, about the baby. You know -- I did feel like that, but it was more of a process -- I'm not saying it was but that was how it felt at the time the process to get this baby born today rather than staff having to hang on’.*

When asked why she went along with the suggestion Paula said that ‘you’ (women) did not question things then and as a ‘*medical professional’* had told her what was going to happen the assumption was ‘*you need this and that’s it’.*

What emerged from the data overall was a strong sense of understanding as acceptance. Heidegger helps us to understand such passivity explaining how in its *‘everydayness’* Dasein is ‘*disburdened*’ by the *‘they’*; the ‘*they*’ make every choice and decision meaning that Dasein assumes a passive role and, in so doing, is disburdened of moral responsibility and autonomy [27].

The majority of the women talked of birth as an overwhelmingly managed experience, as a consequence of pregnancy and a gateway to motherhood, with Meg saying that she did not really have any idea what being pregnant or giving birth was like but she figured that ‘*it was just something somebody did when they got married’.* Likewise Marie said ‘*I don’t think I ever questioned what it was like….we were going to get a child at the end’.*

Certainly for many of these women a positive experience was measured by things turning out ‘*alright’* and being able to take home a healthy baby. Paula explains, ‘*I had two babies and everything was alright, so they were positive experiences for me’.* Sophie was of a similar mind-set saying that, ‘*you go into it thinking all I want really is a healthy baby’.*

#### ‘This generation nothing’s private to them’

The women birthing in the 1970s-80s spoke at some length about the fact that birth was a ‘*pretty private thing to talk about’* (Sandra). Likewise the women birthing in the present day said that they had not always felt comfortable speaking to their mothers or grandmothers about birth and that their mothers had not necessarily wanted to speak about birth with them. For some it was a cultural issue; Rebecca, for instance, was of Chinese origin and was born in Hong Kong. Her grandmother was educated in a very traditional manner and did not feel it was appropriate to discuss childbearing with her granddaughter. Similarly Lucy, also Chinese, expressed a similar view saying that ‘*they can’t really talk about that in the past….it’s just a cultural influence. They just found that it is something very private, something quite embarrassing to talk about’.*

Meg said that she did not remember her mother saying very much about pregnancy and reasoned ‘*I think it was inappropriate to go into too much detail because, you know, genitals weren’t something you referred to in those days’.* For others it was simply not something they talked about; Emma said that she had not really spoken with her mother about birth and did not feel she was in the position to ask her mother about birth saying ‘*I’m sure it’s a generational thing. My mum wouldn’t have been as open about things as perhaps I would be with my daughter’*.

Sandra commented that when she was pregnant she worked with women who had children but that ‘*we never discussed what it was like. It was different then to how it is now’*. Sandra felt that it was a *‘pretty private thing to talk about’* and that young women ‘today’ talk about birth more than her own generation or the generation before that saying ‘*in this generation nothings private to them - nothings off limits, they talk about everything’*.

#### ‘Information seeking and saturation’

The women birthing in the present day were searching for information on which to base their choices related to childbearing and as such they pursued many story mediums. There was a sense that they needed to ‘research’ birth much as you might research a new purchase. Charlotte explained:

‘*I feel like I have to be informed. Just because I’m like that with everything….. I would never just launch myself into something without reading up on it or researching it first’.*

The women appeared overloaded with information amassed from a variety of sources some of which they felt was conflicting (Rebecca) and some of which they weren’t sure was authoritative and therefore to be relied on (Mary). Stephanie, for instance, spoke about the fact that the more she read the more confused she got until she felt ‘*I just really don’t know what I want to know because I just think well, I don’t know now*‘. Many of the women reached a point where they were no longer open to information. Stephanie was very clear describing how she told her husband:

*‘I don’t want anything more because I’ve got to the point where I’ve reached saturation....I’m not buying any books. I’m not getting any in because I’ve just reached overload that I don’t actually know what is going on in my head’.*

Nearly all the women birthing in the present day relied heavily on the internet as a means of accessing birth stories and as a source of information. Mary talked about using it *‘where I need quick answers on things’* and Joanna suggested that it is a useful tool ‘*if you’re having an ‘am I allowed to take Rennies or not’ moment’*. For some the internet was not merely an information source but was also a place to access social support in the form of online communities. Charlotte explained:

*‘You can talk about anything on there, like if you're worried about birth or whatever and people have exactly the same sort of questions that I have‘.*

Despite the value many of the women placed on the virtual community some were quite sceptical about it. Mary got to the hub of the matter stating, *‘I mean you don’t know who they are. You don’t know whether it’s true. It might not be helpful. It might just scare you’.* For Mary the internet could be a dangerous place as *‘everybody’s an expert’.*

In his account of technology Heidegger maintains that in the modern world *things* reveal themselves to us ‘technologically’; that is they reveal themselves as resources for our ends. Heidegger explains that practices in this technological world come to be favoured in terms of their performance, according to some standard of efficiency, and that these standards provide the ultimate criterion for deciding on a course of action [27].

In this study there was a sense that women appropriated the internet and integrated it into their experience of pregnancy and childbirth, using it to help them make choices and decisions. There was almost a sense of them having to use the resource because it was available; when Mary was asked why people accessed the internet for information (even when they knew it wasn’t always reliable) she said:

*‘Oh because you can, it’s there. Yeah. I mean it’s an absurd world we live in; you can key in a question and get an answer to anything. You just don’t know whether it’s right’.*

#### ‘Birth in the twilight of certainty’

This theme examines women’s experience of *Being* in a system of birth as constructed, portrayed and sustained in the stories being shared. The sub-themes ‘*on the conveyor belt of care’*, ‘*birth as a technological feat’* and ‘*being a good patient and a good parent’* explore women’s experiences of this world and the responsibility and pressure they feel to behave appropriately; conforming to accepted conventions and, in so doing, further sustaining and ultimately propagating the ‘modern birth story’.

#### ‘On the conveyor belt of care’

A number of the women discussed the notion of being part of a ‘*system*’ of birth suggesting that they felt like one of the ‘*processes*’. Meg who gave birth in the 1970s-1980s, said that:

‘*I just felt like one of those processes…… your job was to produce this baby…it was about getting the baby out’.*

Jean, who gave birth in the same era, said of her first birth. ‘*I seemed to be just pushed from pillar to post on this kind of never ending conveyor belt’.* For Meg being part of the system was a frightening experience as ‘*nothing was explained’* and she did not feel that the people ‘caring’ for her were concerned about her welfare.

For Ruth, however, who was pregnant in 2012 with a much wanted baby after fertility treatment, birth was merely another ‘*process’* she had to go through to have her ‘*dream baby’*. Up until this point Ruth’s path to having a baby and becoming a mother had been keenly managed; any sense of uncertainty had been removed from the experience and Ruth felt ‘*in control’* of what was happening. Being part of the ‘*process’* of childbirth similarly reassured her and helped her maintain that feeling of control.

Joanna, pregnant in 2012, spoke at some length about her experience of the ‘*system*’ of birth saying that when ‘you’ (women) get pregnant there’s an assumption that you’ll do all the ‘*routine things’* (like have ultrasound scans and screening) even though some of them are ‘*supposedly optional’*. Joanna remarked:

*‘It’s a bit like being on a conveyor belt and actually if you do nothing it’s just going to happen anyway. You turn up and you’re in the system and you just sort of potter along, going along to the next appointment when you have to’.*

For Joanna being in the ‘system’ might have felt a little impersonal but was ultimately reassuring because:

‘I*t’s just so routine; you know what you’re meant to be doing and you know what you’re meant to be finding out and that they will check various things to make sure you’re still well’*.

Joanna was reassured by the routine nature of the antenatal care; she was part of a system like every other woman and if there was anything to worry about she would need ‘*special treatment’*. As part of the ‘*process*’, Joanna was conforming to the social norms of care as shared in the stories she had seen and heard and, in doing so, disburdening herself of the need to make difficult choices and decisions.

Heidegger explains that the notion of ‘care’ is central to our being-in-the-world describing how the world can be defined as what we care for, and we can be defined as what cares for the world [26]. As care we *have* care and we *take* care. It is through care that we are able to understand ourselves and our existence. Care is the means by which facts, possibilities, people and events in the world matter to us. The world described by the majority of the participants in this study is one where caring involves ‘*leaping in’* and ‘*dominating*’; health professionals taking up the burden of care and managing women’s births for them [27].

#### ‘Birth as a technological feat’

Jean, who gave birth in the 1970s-1980s, said that when she was pregnant she felt birthing was a ‘*natural thing to do’* explaining that she was a ‘*no-fuss’* kind of person who did not anticipate complications. Speaking of her daughter’s experiences of birthing Jean said that she felt her experiences were made more complex by the volume of information available (circulated via the various storying mediums) and the technology relied on to *‘monitor’* maternal and fetal wellbeing.

Jean spoke of the world of birth now as *‘high tech’* saying she understood the need for technology from ‘*the safety point of view*’ and yet she felt uncomfortable with women being ‘*attached to all these wires and goodness knows what else*’. Jean’s language suggests that to ‘succeed’ in birth today women must yield to and exploit the technology surrounding it.

Conversely Sandra, who also gave birth in the 1970s-1980s, did not appear to think that birth was now more medicalised than when she birthed saying of her first birth:

*‘They had to break my waters to bring it on quicker because the waters weren’t breaking. I can remember having the waters broken. I could remember they put like a little clip on her head and I think that was so they could hear her heartbeat….. I could remember they put a belt around me which monitored the contractions’.*

Sandra’s own experiences of birthing were medically managed and for her this was clearly the norm; as a result the ‘modern’ landscape of birth portrayed by a myriad of assorted story mediums did not look very different. However for Paula birthing ‘technology’ such as ultrasound scans and electronic fetal monitors made the landscape of birth more complex but paradoxically had not necessarily improved women’s experience, saying of her daughter:

*‘She had a horrendous, horrendous time. And when she was actually delivering -- she had to go in for an epidural and she had -- because of all this pain she had with the pelvis and then she had a really bad reaction to the epidural. I mean I wasn't there – Paul her husband was with her and he actually thought she was going to die because she was out, you know, during that.’*

Paula was shocked that despite all the ‘preparation’ her daughter had done in the form of information gathering, and despite what she perceived to be ‘improvements’ in care (such as routine ultrasound scans) her daughter’s experience had been more negative than her own (when the information wasn’t as widely available and the technology not as well advanced).

For a lot of the women birthing in the present day there was an expectation that birth would be medically managed; for some this was because they had health issues (Harriet had a heart condition and Mary had had previous major abdominal surgery), for others, as discussed earlier, it was something they anticipated from the stories they had heard and the representations of birth they had seen (Stephanie and Isabel).

The experience of being-in-the-world of birth for these women was an experience of being in a world populated by doctors and technology; all in place to safely ‘manage’ their well-being and their births. Heidegger’s concepts of ‘*facticity*’ and *‘ruinance’* help us understand this; Heidegger’s view is that the human way of being is incomprehensible in isolation from a grasp of the world in which it ‘is’ [27]. Dasein exists in an environment in which it is ‘*tempted, seduced, soothed or estranged’* by the world around it [34]. The childbearing woman then can never just ‘be’ within the world of birth without already being a part of it and potentially being ‘spoiled*’* by it.

Being ‘spoiled’ by the modern technological world is something which concerned Heidegger as he believed that technology held more danger than potential and had the capacity to obscure the meaningful presence of things; he spoke of the modern world as a world where things show up as having the potential to be ordered according to the norms of control and efficiency of that world [35]. In this world people share a way of ‘being’ with all other ‘things’ and are therefore prized in terms of their ability to function as another ‘resource’; to be productive and efficient.

The notion of birth as a ‘*technological feat’* in which women are tasked with yielding to and exploiting technology is a disturbing one; in this interpretation women’s disembodied experience of birth is accepted as normal and mainstream. The problem with a meaning that supposedly increases the *‘orderability’* of birth and utilises calculative thought is that it sees women as standardised resources with reproductive capacities.

#### ‘Being a good patient and a good parent’

Women birthing in the present day spoke about the responsibility to behave as a ‘good patient’ whilst pregnant and birthing, and perform as a ‘good parent’ both in relation to their developing foetus and newborn baby. Stephanie spoke about her previous experiences of attending hospital for operations and the ‘expected’ behaviour she would conform to; being told where to go, getting changed into a hospital gown and ‘allowing’ health professionals to do everything for her. Her expectation was that she would do the same in pregnancy saying that the ‘*professionals will probably tell you - we want you like this’.*

For Isabel being a ‘*good patient’* involved ‘*hopping up on the bed’, ‘lying still and being good’* and not ‘*making a fuss’* or being a ‘*nuisance to anyone’*. Isabel said that she always wanted to please people and that when she went on to give birth she would be particularly anxious to please. Being a ‘good patient’ proved problematic however when Isabel attended the hospital for a glucose tolerance test; Isabel said she ‘*wanted to do well’* but that it was the hardest 2 hours of her life as she felt so violently sick. Isabel did not want to ‘*ruin the test’* which she felt was crucial to being a good patient but also significant in being a good parent as she ‘*wanted to have the test to make sure that everything was fine*’ with the baby.

Childbearing women in the modern world of birth are faced with an endless array of both expert and lay advice (much of it shared in the ‘modern’ birth story) about the ways in which they should protect their foetuses and babies from risk and promote their health and wellbeing. This ‘pressure’ to make the right choices and to fit the profile of the ‘perfect mother’ is encapsulated by what Isabel describes as the ‘*massive list of rules about your baby’*. Isabel gave examples such as the need to keep doctor’s appointments whilst pregnant, the responsibility to get the baby vaccinated, and the necessity to ensure the baby sleeps in the ‘correct’ position and is covered by the right number of blankets. Isabel was undoubtedly anxious that she fit the requirements of a ‘*good parent*’ saying that she strove to be what she described as a ‘*good vessel’* for her baby, by doing everything in her control to ‘*help the baby*’ and ‘*protect it’*; in this sense she was endeavouring to ‘*tick all the boxes and get it all perfect’.*

Isabel’s desire to please everyone and her responsibility to protect her baby (from a potentially dangerous birth and possibly from her own poor decisions because she is not an ‘expert’) suggested that she would seek out guidance and care which absolved her from responsibility and instead put others who had the necessary expertise in charge.

Similarly at antenatal classes Lucy learned she could make decisions about what is ‘*right for her and her baby’*. Doing what is right is a responsibility and Lucy talked to friends, watched the television and read books to try and get ready for the experience. Lucy believed that knowledge was power and that being informed would alleviate some of her fears about birth, helping her to have the experience that she wanted but ultimately helping her to make the right choices and decisions which would have the best possible outcome for her baby.

## Discussion

Birth stories are cultural ‘productions’ that convey various ideologies and belief systems shaping women’s expectations and experience of childbirth. Rather than merely reflecting existing ideas and values, the stories women tell embody the values and belief systems of our society and, in so doing, ‘*colonize consciousness’* and ‘*come to constitute and sustain the lifeworld’* of birth [36].

This study showed that the information gleaned from birth stories did not create meaningful knowledge and understanding for women; thrown into the world of birth the women were similarly thrown into the dialogue of that world and the dialogue spoke of intervention, management and an increasing reliance on technology. The ‘idle talk’ shared shaped women’s expectations of birth and determined the way that they expected to ‘perform’ birth.

For the women who gave birth in the 1970s-1980s a positive birth was characterised exclusively by the birth of a healthy baby; the journey towards which was a stepping stone to becoming a mother and an experience managed by the experts who were in place to help keep them safe. For the women who were pregnant in 2012 there were a myriad of different mediums available to help them prepare for birth and ensure they were able to make the ‘correct’ choices ensuring the health of their unborn baby and demonstrating their competency as mothers.

Both groups of women understood care givers as holding the key to ‘*safe passage’*; with the ability to protect them from the ‘risks’ associated with birth and the knowledge to help them navigate choices to ensure a controlled, predictable outcome [37, 38]. Neither group was comfortable with the notion of uncertainty wanting the guarantee of the ‘perfect baby’ and seemingly prepared to forgo their autonomy in their quest to achieve that end. Despite framing their expectations differently, in the language of safety (for the older generation) and choice (for the younger), the world of birth was the same for both groups of women; saturated with, and only legitimised by the birth of a healthy baby.

In a context where choice in childbirth is increasingly considered paramount it is disturbing (but not surprising) that choice in this study was synonymous with safety. The study findings reinforce the notion that women’s agency and choice in birth is limited; ‘*restricted by protocols, hierarchy and fear*’ [39] and that a reliance on medicalisation and technology to guarantee safety both hinders and standardises choice [38].

For both groups of women the stories shared persuaded them that birth was a ‘drama’ to be navigated and forgotten. Seeking sanctuary from the ‘drama’ of birth many of the women persuaded themselves they would be more secure within the system of birth where accountability rested with the experts. Both groups of women chose to accept the birth practices around them; experiencing themselves and their bodies as part of the wider machinery of birth rather than coping with uncertainty and taking responsibility for the consequences [40].

### Limitations

A number of limitations are apparent in this study and may therefore affect the usability of its findings. The first is that the participants were all from England and the birth stories they heard and told were shared within the context of the prevailing maternity system. Whilst the phenomenological descriptions of birth stories outlined in this study may be shared by women in other high resource settings with similar models of maternity care and societal and cultural norms, they are unlikely to be replicated in contexts where the models of care and socio-cultural norms are fundamentally different.

The women were recruited from a particular socio-economic demographic; most were white Caucasian and were from a largely from a middle class background. It was likely therefore that these women viewed the world through a lens skewed to their way of thinking and their way of being in the world.

Finally LK’s ‘immersion’ in the study may be considered a limitation; however this research does not pretend to be objective. In hermeneutic phenomenology the researcher’s understandings are an intrinsic part of the interpretive process [25]. As Crowther et al. explain a story’s ‘*truthfulness*’ (described as ‘*unconcealedness*’) is known to us by how it ‘*resonates in felt, shared plausible meaning, and this resonance cannot be reified into proof*’ [25]. A dynamic entity which changes and takes different forms as different influences are added, the interpretation presented is a never ending process, a process which relies on the reader to add their own ‘layer’ to the interpretation.

## Conclusion

Our findings suggest that women birthing today although able to access a huge array of information about childbirth, are not well prepared for birthing their babies. Rather they are overloaded with information sought in an attempt to prepare for the unexpected, address their anxiety and demonstrate their competency as mothers. Further these women are delimited by the ‘idle talk’ surrounding birth; which serves to emphasis the hype of birth as opposed to giving them any real understanding of birth and/or creating meaningful knowledge.

Moving forward women need to be encouraged to seek out and share positive stories and be told how powerful these stories can be in reinforcing women’s capacity to birth. Women must be given the tools to appreciate the potential of birth to be something other than a drama in today’s childbearing world. The nature of the idle talk being shared around birth needs to change so that the default story is not impersonal, perilous and out of place.

This study highlights a need for further research to qualify the relationship between what women see and hear about birth and their consequent expectations and experiences. Further it demonstrates that work is needed to ensure stories and media portrayals of birth support women’s confidence in their capacity to birth positively in a range of different circumstances.

## Abbreviations

CBN: Cambridge Business women’s Network
NCT: National Childbirth Trust
NFWI: National Federation of Women’s Institutes
STEMH: Science, Technology, Engineering, Medicine and Health Ethics Committee
